# A cluster randomized controlled trial of a community‐based initiative to reduce stunting in rural Indonesia

**DOI:** 10.1111/mcn.13593

**Published:** 2023-12-02

**Authors:** Amanda Beatty, Evan Borkum, William Leith, Clair Null, Wayan Suriastini

**Affiliations:** ^1^ Youth Impact Garorone Botswana; ^2^ Mathematica Inc Washington DC USA; ^3^ SurveyMETER Yogyakarta Indonesia

**Keywords:** community‐driven development, community‐led total sanitation, Indonesia, stunting, undernutrition

## Abstract

We evaluate the impacts of a $120 million project in Indonesia conducted between 2014 and 2018 that sought to reduce stunting through a combination of (1) community‐driven development grants targeted at health and education outcomes, (2) training for health providers on infant and young child feeding and growth monitoring and (3) training for sanitarians on a local variation of community‐led total sanitation. This cluster randomized controlled trial involved 95 treatment and 95 control subdistricts across South Sumatra, West Kalimantan, and Central Kalimantan provinces. Overall, we find no significant impacts on stunting, the study's primary outcome measure (0.5 pp; 95% confidence interval [CI]: −3.0 to 4.1 percentage points [pp]), or other longer‐term undernutrition outcomes about 1 year after the end of the project. The project had a modest impact on some secondary, more proximal outcomes related to maternal and child nutrition, including the percentage of mothers consuming the recommended number of iron‐folic acid pills during pregnancy (8.7 pp; 95% CI: 4.1–13.3 pp), 0–5‐month‐olds being exclusively breastfed (8.7 pp; 95% CI: 1.8–15.6 pp) and 6–23‐month‐olds receiving the number of recommended meals per day (8.5 pp; 95% CI: 3.8–13.2 pp). However, there were no significant impacts on other proximal outcomes like the number of pre‐natal and post‐natal checkups, child dietary diversity, child vitamin A receipt or the incidence of child diarrhoea. Our findings highlight that successfully implementing an integrated package of interventions to reduce child stunting may be challenging in practice. Project design needs to consider implementation reality along with best practice—for example, by piloting the synchronous implementation of multifaceted interventions or phasing them in more gradually over a longer timeframe.

## Key messages

- A well‐resourced multifaceted project that sought to reduce stunting through (1) a health and education‐focused community‐driven development programme, (2) provider training on infant and young child feeding and growth monitoring, and (3) training on community‐led total sanitation had no impact on child stunting rates although it successfully improved aspects of maternal nutrition during pregnancy, exclusive breastfeeding, and complementary feeding.
- Successfully implementing an integrated package of interventions to reduce child stunting may be challenging in practice, especially in a typical programme timeframe.
- Piloting the synchronous implementation of multifaceted interventions or phasing them in more gradually are potential solutions but might require funders to commit to longer implementation timeframes.

## Graphical abstract

We conducted a randomized controlled trial of a community‐based maternal and child health project in Indonesia. Overall, we find no significant impacts on stunting, the study's primary outcome measure or other longer‐term undernutrition outcomes. The project had a modest impact on some secondary, more proximal outcomes related to maternal and child nutrition.

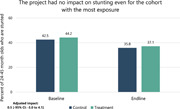

## 1 INTRODUCTION

Child stunting affected an estimated 35% of children under 5 years old in rural areas of Indonesia and 27% in urban areas of the country in 2018, according to the latest Indonesia Basic Health Research (RISKESDAS) survey (Kementerian Kesehatan, 2018). Given that Indonesia is the world's fourth most populous country, it has consistently accounted for about 5% of the global stunting burden among children of this age group over the past decade (United Nations Children's Fund UNICEF et al., 2021). Reducing child stunting has increasingly become a policy priority for the Indonesian government, which is implementing a national stunting reduction strategy that seeks to increase and coordinate stunting‐related programmes (Republic of Indonesia, 2018). These programmes comprise nutrition‐specific and nutrition‐sensitive interventions for pregnant women and young children, which aim to affect children in the 1000‐day period from conception through age 23 months, a critical period for their growth and development (Karakochuk et al., 2017; Victora et al., 2010).

Globally, community‐based platforms are a promising channel through which to deliver these types of interventions to poor and remote populations (Bhutta et al., 2013). These platforms, which often rely on community health providers operating in the existing local health system, are conducive to delivering nutrition‐related interventions as a package, an approach that has the greatest potential to reduce stunting (Bhutta et al., 2013; Dewey, 2016; Hossain et al., 2017; Mangasaryan et al., 2011). At the same time, broader investments in population health, education, and social development are crucial in providing a supportive context for improvements in stunting and other nutrition‐related outcomes (Bhutta et al., 2013, 2020; Hossain et al., 2017; United Nations Children's Fund UNICEF, 2013).

In Indonesia, a health and education‐focused community‐driven development (CDD) programme, *Program Nasional Pemberdayaan Masyarakat—Generasi Sehat dan Cerdas*, also known as *PNPM‐Generasi* or *Generasi*, was first introduced in 2007. The programme provided communities with grants they could use to achieve progress on the project's 12 health and education service delivery indicators. These included indicators related to maternal and child nutrition, such as distributing iron tablets to pregnant women and regularly weighing infants and young children. A randomized controlled trial of the pilot programme (Olken et al., 2014) found a significant reduction in the percentage of children under 3 years old who were underweight. The study also found no impact on stunting overall, but in one province with the highest baseline rates of poor nutrition, it reduced underweight rates by 9 percentage points (pp) and stunting rates by 7 pp.

In this study, we report on the impacts of the $120 million Community‐Based Health and Nutrition to Reduce Stunting Project in Indonesia, which was funded by the Millennium Challenge Corporation (MCC), a US foreign aid agency, and was implemented in 11 of Indonesia's 34 provinces between 2014 and 2018. The project supplemented a slightly modified version of the original *Generasi* programme with two additional sets of interventions designed to increase impacts on stunting: (1) intensive training for community health providers on infant and young child feeding (IYCF) and child growth monitoring and (2) training for sanitarians, public health workers who encourage healthy sanitation behaviours in communities, on a local variation of community‐led total sanitation (CLTS). The training for community health providers was intended to increase community members' focus on maternal and child nutrition and support communities' abilities to make progress on the relevant *Generasi* indicators. CLTS was included in the project because lack of access to sanitation is associated with higher child diarrhoea (Fink et al., 2011; Headey & Palloni, 2019) and therefore lower availability of nutrients for growth, although evidence on the relationship between sanitation access and stunting is mixed (Fink et al., 2011; Headey & Palloni, 2019; Pickering et al., 2019; Spears et al., 2013). Most project interventions were implemented at the subdistrict level, facilitating a cluster randomized controlled trial at that level.

The primary objective of this study was to assess the extent to which this comprehensive package of community‐based interventions was effective in reducing stunting, the primary outcome measure, among children under 3 years old in the treatment villages. An additional objective was to assess impacts on secondary, more proximal outcomes related to nutrition and sanitation practices.

## 2 METHODS

### 2.1 Study sites

This study took place in three of the 11 provinces in Indonesia in which the project was implemented: South Sumatra, West Kalimantan and Central Kalimantan. These three provinces had population sizes of 7,829,000, 4,641,000, and 2,345,000, respectively, at the start of the project in 2013 (Badan Pusat Statistik, 2013). MCC selected these provinces for the study because they were among the country's 10 provinces with the highest stunting prevalence, had not already received *Generasi* through other funding sources, and had enough potentially eligible subdistricts for the study (the unit of random assignment). The project targeted 22 districts in these provinces that had the highest child stunting rates, lowest rates of health and education access and use, and lowest rates of 'supply readiness' (based on the number of health and education facilities, quality of health and education infrastructure, and community characteristics). Within these 22 districts, the project identified the 234 (out of 283) subdistricts that were participating or expected to participate in a government programme targeted at rural communities; willingness to participate in this programme was another sign of readiness for the project.

### 2.2 Study design and random assignment

**Figure 1:**
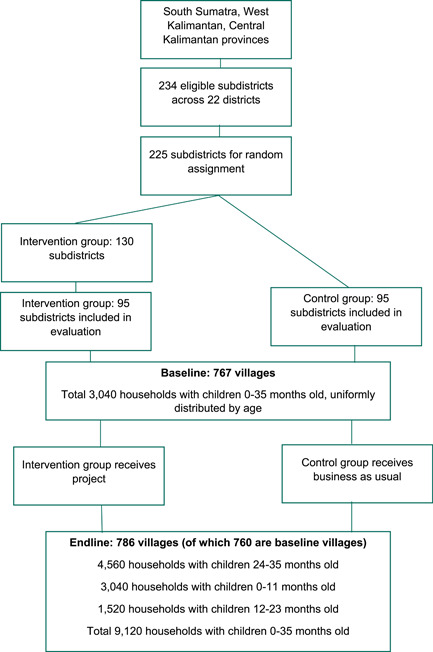
Study design and sample.

This study used a clustered randomized controlled trial to assess impacts, with random assignment at the subdistrict level. The random assignment procedure involved three stages: (1) randomly selecting 225 of the 234 eligible subdistricts for random assignment, (2) randomly assigning these subdistricts into 130 treatment and 95 control subdistricts and (3) randomly selecting 95 of the treatment subdistricts for inclusion in the study (Figure 1). The total number of treatment subdistricts (130) was determined based on project implementation targets, whereas the number of control subdistricts (95) was determined based on anticipated sample size needs and attempting to limit data collection costs. An implicit stratification procedure ensured that the final study sample of 95 treatment and 95 control subdistricts was balanced across study districts. The randomized allocation was coded in Stata version 13.0, using a random‐number seed to avoid any manipulation of the allocation.

### 2.3 Participants and sampling

The population of interest for the study was children 0–35 months old and their caregivers. We focused on this population because children 0–35 months old at the time of endline data collection in early 2019 would have been exposed to the project during at least part of the 1000‐day window, given the project was largely implemented between 2014 and 2018. Because the key project outcomes were focused on populations at a particular stage in life, we used a repeated cross‐sectional approach, drawing a different random sample of children 0–35 months old at baseline and endline.

To identify the villages for household sampling, we randomly selected four villages in each treatment and control subdistrict as the primary sampling units (PSU), a total of 760 villages. In a small number of subdistricts, we added an additional village at baseline because the original four were insufficient to meet sample targets, resulting in a total of 767 villages in the baseline sample. All children 0–35 months old and their caregivers in the sampled villages were eligible for the study sample. The original 760 villages selected at baseline remained the PSUs for the endline data collection. However, we added 26 randomly sampled villages at endline to bring us closer to sample size targets, resulting in a total of 786 villages in the endline sample. To mitigate the costs of conducting a household listing in large villages, if the population of a sampled village was larger than a preestablished cutoff based on sample size targets (250 households at baseline and 400 at endline), we determined the appropriate administrative level to use as the secondary sampling unit (SSU) and developed a list of these SSUs with the help of the village head. We then randomly selected one SSU in each PSU; in a few cases, we selected an additional SSU because the selected one was insufficient to meet sample size targets. The baseline and endline surveys were therefore conducted mostly in the same villages (PSUs) but in different SSUs.

Because many expected synchronicities in project activities did not materialize due to implementation delays, project exposure varied substantially across cohorts of children. The primary sample of interest comprised children who were 24–35 months old at endline, because they would have received the most exposure to the project activities in the 1000‐day window, as we discuss below. The endline sample therefore included 4560 children 24–35 months old at endline, which we complemented with a smaller sample of 3040 children 0–11 months old (to capture pregnancy and newborn care behaviours, which were unlikely to be accurately recalled for older children) and 1520 children 12–23 months old (to round out the sample of children to cover the full 0–35‐month age range with at least partial exposure). This amounted to a total sample size of 9120 households (about 12 per village). We estimated that this sample size would enable us to be able to detect stunting impacts of about 5pp or greater for the full 0–35‐month range (6pp for the 24–35 age group) and impacts on behavioural outcomes ranging between 2 and 9pp for the 0–11‐month cohort. These statistical power calculations used estimated means and intra‐cluster correlation coefficients from the baseline survey. The baseline sample size was lower; 3040 children were uniformly distributed over the 0–35‐month age range because we used it only to assess baseline equivalence and provide controls for the endline impact analysis.

### 2.4 Data collection and response rates

Baseline data collection took place from November 2014 to February 2015, when most project activities were still in the planning or early implementation stages. Endline data collection took place from January to April 2019, about 1 year after the end of the project. Within the selected PSUs and SSUs, enumerators from the data collector SurveyMETER conducted a listing of households and drew a simple random sample of households with children in the relevant age range from this listing. If a household had more than one child 0–35 months old, one of the eligible children was randomly selected as the index child for sampling and survey purposes. If a sampled respondent was not successfully interviewed after three attempts, SurveyMETER randomly selected a replacement respondent with a child in the same age range as the sample respondent to meet the sample size targets.

In sampled households, SurveyMETER enumerators interviewed the household head (or another household member who was knowledgeable about the household, if the household head was not available) and the sampled child's caregiver. Survey participants were told that they were being interviewed for a study of maternal and child health in their village, without mentioning the randomized controlled trial. The household head survey included questions about the background characteristics of household members, dwelling, assets, receipt of social benefits, and water and sanitation conditions. The caregiver survey included questions about maternal and child health outcomes such as health care utilization during and after pregnancy, breastfeeding and complementary feeding, and knowledge of caregiving practices. Instrument items were drawn from previous surveys whenever possible, such as the Indonesia Family Life Survey (IFLS), UNICEF Multiple Indicator Cluster Surveys (MICS) and indicators recommended by the World Health Organization WHO (2008).

Each field team included a supervisor, three household enumerators, and two anthropometrists, plus one enumerator for the health provider surveys described below. SurveyMETER provided 12 days of training to the enumerators (185) and anthropometrists (75). Anthropometrists had to pass a standardization test assessing the precision and accuracy of their techniques before being accepted into the data collection team. The anthropometrists conducted length and weight measurements for the sampled children using Seca brand scales and measuring boards. Per WHO protocol, children under 2 years old were measured for length lying down and children over 2 years old were measured standing. All measurements were conducted by two anthropometrists, and if the measurements were substantially different (more than 0.7 cm for length and more than 100 g for weight [De Onis et al., 2004]), both would redo their measurements up to two more times. Two nutritionists were engaged as consultants to observe the data collection activities conducted by SurveyMETER and provide feedback to ensure that all anthropometrists were collecting high‐quality data. The research team also regularly ran a series of data quality checks, including looking for digit preference, the frequency of disagreement between the two anthropometrists on each team, and the distribution of each anthropometrist's measurements.

The household head and caregiver surveys were typically administered during the same visit as the anthropometric measurements, face‐to‐face in the respondent's home. These questionnaires were administered using Computer‐Assisted Personal Interview software, using laptops to enter data during the interview. The supervisor checked the entered data on a daily basis for completeness and consistency. In addition, the supervisor listened to recordings of the interviews to identify any problems with the interview process. The overall endline response rate was about 90% for household heads and caregivers and was almost identical in the treatment and control groups.

In addition to household‐level surveys, SurveyMETER administered surveys to 2405 health service providers in the study areas, which included (1) village‐level health post volunteers and midwives and (2) subdistrict health centre‐level midwife coordinators, nutritionists, and sanitarians. These surveys focused primarily on capturing indicators of project exposure and implementation, such as participation in training and health care practices. SurveyMETER attempted to interview all of these types of providers located in the study areas and achieved a response rate of close to 100%.

### 2.5 Intervention

The project was designed to deliver a comprehensive package of community‐level interventions that together would reduce child stunting. The CDD component of the project, *Generasi*, was expected to create demand for health services among pregnant women and caregivers of infants and young children by enabling communities to choose maternal and child health projects relevant to community needs. *Generasi* provided villages facilitation support and annual grants of about $5000 per year, on average, over 3.5 years (mid‐2014 through 2017). We examined compliance with random assignment by interviewing village administrators in endline sample of villages. 97% of villages in treatment areas reported that they participated in *Generasi* compared to just 2% in control areas. Grants were allocated to villages based on the number of pregnant women and children in each village, the difficulty of accessing education and maternal and child health services, and, after the first year, the progress made during the previous calendar year on 12 health and education indicators established by the project. These indicators included consumption of iron tablets during pregnancy, regular weighing of and weight increases for young children, vitamin A consumption by children, and participation of pregnant women and mothers of young children in group nutritional counselling sessions (Table A1).

In practice, communities spent more than two‐thirds of total *Generasi* funds on health‐related purposes (Beatty et al., 2020). The largest single category of spending, which amounted to a quarter of *Generasi* funds spent on health, was on in‐kind food assistance for households with pregnant women or children under 5 years old through an existing national programme. Other common activities funded by *Generasi* were incentives for community health providers (usually including transportation funds), health and nutritional counselling activities (transportation funds for patients, along with snacks offered at group counselling sessions), additional training for community health providers on IYCF and growth monitoring, equipment for monthly weighing clinics, and infrastructure support for water and sanitation.

*Generasi* was complemented by two other main groups of interventions. The first group of interventions supported community health workers serving pregnant women and mothers of young children in the communities where *Generasi* was implemented. The project provided training on IYCF and child growth monitoring for health providers (village health post volunteers, village midwives, nutritionists and midwives at subdistrict health clinics, and district and provincial health officials) in these communities. Training lasted between 3 and 8 days, depending on the type of staff targeted. The implementer adapted training content from a UNICEF training and covered a variety of technical topics related to breastfeeding, complementary feeding, growth monitoring, and women's nutrition. Service providers were able to apply what they learned in training in regular village‐level nutritional group counselling sessions for pregnant women and for caregivers of children under 5 years old held in the community, as well as in one‐on‐one counselling and services. The project provided anthropometric kits to the subdistrict‐level health clinic—including length‐ and height‐taking equipment, scales and measuring tapes to measure middle‐upper arm circumference for pregnant women—to help health providers apply their project‐funded training on growth monitoring. It also distributed a WHO‐approved improved formulation of iron folic acid (IFA) to pregnant women, which was designed to reduce side effects and improve flavour compared to the previous formulation, through trained community health workers.

These project‐funded interventions were only available in treatment subdistricts, but exposure varied across villages. In provider surveys, 70% of subdistrict health centre‐level midwife coordinators and nutritionists in treatment areas reported having been trained in IYCF during the project, compared to 35% and 43% in control areas who received business‐as‐usual IYCF training. At the village level, 60% of health post volunteers and midwives in treatment areas reported that they had received IYCF training, compared to 30% in control areas. 35% of subdistrict health centre‐level midwife coordinators and 58% of nutritionists in treatment areas reported having been trained in growth monitoring during the project, compared to 18% and 44% in control areas, respectively. For both types of training, project‐funded training tended to be longer, covered more topics and used more innovative teaching approaches than business‐as‐usual training. 57% of mothers in treatment areas who consumed IFA tablets at endline received the IFA brand supported by the project, compared to 12% in control areas.

The second group of interventions sought to improve sanitation in the communities where *Generasi* was implemented by eliminating open defecation. The project provided CLTS training for sanitarians based at the subdistrict health clinic and community volunteers. CLTS seeks to build awareness about the health and safety risks inherent in open defecation, invoke a sense of disgust and shame among community members through a communal event (called triggering), and help communities engage in joint decision‐making and planning to become open defecation free (ODF). The hope was that triggering would motivate villages to improve sanitation behaviour and hygiene practices—for example, constructing a latrine and no longer practising open defecation. This behaviour change was expected to lead to a reduced incidence of diarrhoea and worm infestation and the associated loss of nutrients. According to the sanitarian survey, 81% of sanitarians in treatment areas were trained in CLTS over the project period. Other organizations provided CLTS training in control areas, where 57% of sanitarians were trained, but project‐funded training was longer, more likely to include messages about stunting, and used more interactive teaching methods.

**Figure 2:**
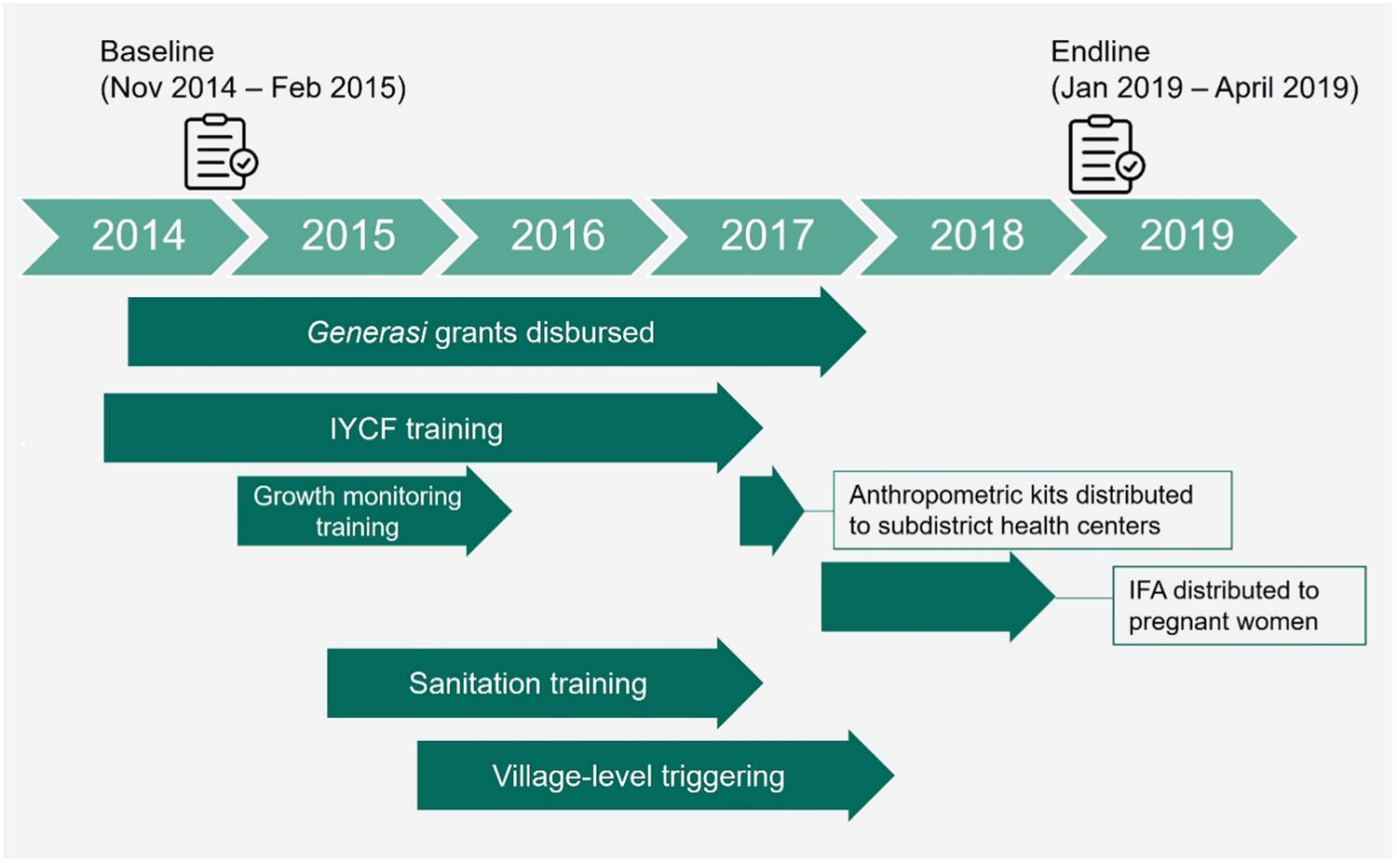
Project implementation and data collection timeline. IFA, iron and folic acid; IYCF, infant and young child feeding.

All activities were to be synchronized to have the maximum impact by affecting children for the full 1000‐day period from conception through age 23 months. However, this synchronicity was a challenge for project implementation. *Generasi* was largely implemented on time, but most other activities—IYCF and growth monitoring training, anthropometric kit distribution, IFA tablet distribution and CLTS training—were implemented later than planned. (Figure 2 shows the full implementation timeline.) Nevertheless, most implementation targets were ultimately met or nearly met, except for the village triggering and ODF targets under the CLTS component (Beatty et al., 2020). Given delays in implementation, the cohort of children 24–35 months old at endline (born between early 2016 and early 2017) would have received the most exposure to the project activities. Specifically, within the 1000‐day window, these children would have been exposed to Generasi implementation in utero and for up to 2 years after birth, IYCF training for between 1 and 2 years after birth, growth monitoring training (albeit not anthropometric kits) for a full 2 years after birth, and triggering for up to 2 years after birth. However, they would not have benefitted from the effects of IFA provision to their mothers during pregnancy, as these pills were only distributed to pregnant women starting in late 2017.

### 2.6 Analysis

We assessed impacts on stunting, as well as on proximal matenal, newborn, and child health (MNCH) and sanitation‐related outcomes through which the project expected to reduce stunting, by comparing endline outcomes between the treatment and control groups using a multivariate ordinary least squares model to conduct an intention‐to‐treat analysis. The model controlled for district indicators (the level of implicit stratification) and individual socio‐demographic control variables related to MNCH outcomes (whether the respondent completed junior secondary school, wealth quintile, mother's age when the sampled child was born, birth order of the sampled child and the sampled child's gender). To assess differences in baseline characteristics and outcomes, we used a similar approach except without the socio‐demographic controls.

In the case of binary outcomes, which include stunting, this approach amounted to a linear probability model. Although probit or logit models are often used for binary outcomes, we prefer a linear probability model because it is easier to interpret and relies on weaker parametric assumptions. In practice, probit or logit and linear probability models generally yield similar results for the types of marginal effects that we estimate here (Angrist & Pischke, 2008; Wooldridge, 2010).

Because the sample of interest consisted of children in a specific age range (0–35 months old) at the time of the survey, we measured key outcomes for different individuals at baseline and endline. Therefore, individual‐level baseline measures of the outcomes are not defined for the endline sample. Instead, in the endline analysis, we adjusted for the baseline mean of the outcome at the subdistrict level—the level of random assignment—by including this mean as an additional control variable. Conceptually, this is similar to a subdistrict‐level difference‐in‐differences approach but is a more flexible approach that allows the relationship between the endline and baseline means to be determined empirically. Using these control variables enabled us to adjust for any baseline differences between the treatment and control groups that arose by chance and improved the precision of the estimates.

At both baseline and endline, we accounted for clustering at the subdistrict level, the level of random assignment, by estimating cluster‐robust standard errors. All analyses included the full set of 95 treatment and 95 control clusters, but individual‐level sample sizes vary across outcomes depending on relevance by child age and item nonresponse. In addition, the implicit stratification approach we used for random assignment led to slightly different random assignment probabilities across the subdistricts in the study. We therefore included weights at baseline and endline to adjust for the different random assignment probabilities, as well as different sampling probabilities at the PSU, SSU and household level. We conducted all analyses in Stata version 15.0.

### 2.7 Ethical considerations

For baseline, the study was approved by the Ethics Committee of Faculty of Medicine at the University of Indonesia in September 2014. For endline, the study was approved by the Health Media Lab Institutional Review Board (IRB) in January 2019, the SurveyMETER IRB in December 2018 and the Universitas Atma Jaya IRB in January 2019. Before collecting any data, verbal informed consent was obtained from all respondents.

## 3 RESULTS

### 3.1 Treatment and control balance

The demographic and socioeconomic characteristics of the households and children sampled at endline were very similar in the two study arms, with small and statistically insignificant differences in all the characteristics we considered (Table 1). There were also no substantive or statistically significant differences in stunting rates or proximal MNCH outcomes between the two arms at baseline. Although we did not measure all endline MNCH outcomes in the baseline survey, the baseline treatment‐control similarity in the outcomes we did measure, as well as similarity in endline household and child characteristics, confirms that random assignment successfully created equivalent groups for the study.

**Table 1:** Endline characteristics and baseline outcomes.

	Sample (child age) (months)	*N*	Control mean	Treatment mean	Difference^a^	*p*‐Value^a^
Endline characteristics						
Female‐headed household (%)	0–35	9.120	4.9	3.9	−0.8	0.278
Muslim (%)	0–35	9.120	72.4	70.1	0.9	0.759
Number of household members	0–35	9.120	4.9	4.9	0	0.982
Caregiver's age (years)	0–35	3.039	28	28	0	0.838
Sampled child is female (%)	0–35	3.022	50.1	48.7	−1.3	0.526
Sampled child is first born (%)	0–35	3.039	37.7	37.4	0.0	0.993
Household wealth index: lowest quintile^b^ (%)	0–35	9.120	20.0	19.4	−0.7	0.664
Household wealth index: highest quintile^b^ (%)	0–35	9.120	19.9	18.9	−1.1	0.393
Baseline outcomes						
Stunted (%)	24–35	916	42.5	44.2	2.7	0.482
Stunted (%)	0–35	2.979	31.4	32.2	1.3	0.563
At least four pre‐natal checkups (%)^c^	0–11	1.046	69.4	71.3	1.6	0.651
Consumed 90 IFA tablets during pregnancy (%)	0–11	1.026	14.7	12.7	0.3	0.921
At least three postnatal checkups for baby during the first 6 weeks (%)^c^	1.5–11	961	9.5	8.4	−2.0	0.407
EIBF^d^ (%)	0–23	2.065	32.3	35.7	0.5	0.845
Vitamin A twice yearly (%)	6–35	2.440	26.7	29.3	1.0	0.691
Child had diarrhoea in past 2 weeks (%)	0–5	449	6.1	3.6	−1.0	0.157
Child had diarrhoea in past 2 weeks (%)	0–35	2.615	6.8	5.9	−1.7	0.148

Abbreviations: EIBF, early initiation of breastfeeding; IFA, iron and folic acid.

^a^ Adjusted for district. The design effect of cluster random assignment was accounted for using Stata's cluster option.

^b^ We conducted a principal components analysis using very similar items to those used by the Demographic and Health Survey and used the coefficients from the first principal component to create the wealth index for each household. We then divided the sample into wealth quintiles based on the distribution of the index in the control group.

^c^ Measured using maternal and child health books when available or through self‐reports otherwise. The baseline survey asked about visits during the first 6 weeks and the endline survey about visits during the first 6 weeks.

^d^ Other breastfeeding and complementary feeding outcomes analysed at endline were not measured in an equivalent way at baseline and are not reported here.

### 3.2 Anthropometric outcomes

Stunting was the primary outcome measure for this study. In the treatment group, stunting rates for the sample of 24–35‐month‐old children, the age group with most exposure to the project at endline, decreased from 44.2% at baseline to 37.1% at endline. However, the control group experienced a similar decrease, so there were no significant impacts on stunting rates either for this age group (0.5 pp; 95% confidence interval [CI]: −3.0 to 4.1 pp) or for the full sample of 0–35‐month‐old children (0.3 pp; 95% CI: −2.3 to 2.9) (Table 2). There was also no significant impact on height‐for‐age *z*‐scores (HAZ), nor on the percentage of underweight or wasted children, for either age group, secondary long‐term nutritional outcomes that might have been impacted by the project.

**Table 2:** Impacts on anthropometric outcomes.

	Sample (child age) (months)	*N*	Control mean	Treatment mean	Adjusted impact (95% confidence interval)^a^	*p*‐Value^a^
Sample with most exposure						
Stunted (%)	24–35	4.513	35.8	37.1	0.5 (−3.0 to 4.1)	0.774
HAZ (SD)	24–35	4.513	−1.60	−1.66	−0.02 (−0.12 to 0.07)	0.637
Underweight (%)	24–35	4.515	28.3	30.6	1.7 (−1.9 to 5.2)	0.360
Wasted (%)	24–35	4.512	8.8	10.7	1.6 (−0.7 to 4.0)	0.180
Full sample						
Stunted (%)	0–35	9.114	28.2	28.4	0.3 (−2.3 to 2,9)	0.812
HAZ (SD)	0–35	9.114	−1.37	−1.38	0.00 (−0.07 to 0.07)	0.984
Underweight (%)	0–35	9.120	21.6	22.3	0.4 (−1.9 to 2.7)	0.781
Wasted (%)	0–35	9.112	6.8	8.4	1.3 (−1.9 to 2.7)	0.821

*Note*: The village‐level intra‐cluster correlation coefficients based were 0.035 for stunting, 0.060 for HAZ, 0.039 for underweight, and 0.013 for wasted.

Abbreviation: HAZ, height‐for‐age z‐score.

^a^ Adjusted for district, whether the respondent completed junior secondary school, wealth quintile, mother's age when the sampled child was born, birth order of the sampled child, the sampled child's gender and the baseline outcome at the district level. The design effect of cluster random assignment was accounted for using Stata's cluster option.

### 3.3 MNCH outcomes

We also examined impacts on a set of secondary, more proximal outcomes through which the project was expected to affect stunting, including both MNCH‐ and sanitation‐related outcomes. Any impacts on these outcomes did not ultimately translate into impacts on stunting. Nevertheless, identifying specific targeted behaviours that the project was more and less successful in changing is useful to assess where the project logic might have broken down because many of these behaviours have broader value for family and community health beyond their impact on stunting.

The project had a significant positive impact on mothers' self‐reported consumption of the recommended 90 IFA pills during pregnancy (8.7 pp; 95% CI: 4.1−13.3 pp) among mothers of 0–11‐month‐olds at endline, who would have been exposed to the improved formulation IFA tablets distributed through the project (Table 3). There were also modest positive impacts on these mothers having attended any nutritional group counselling sessions for pregnant women (8.4 pp; 95% CI: 2.6−14.1 pp) or caregivers of young children (6.6 pp; 95% CI: 3.7−9.4 pp). However, there were no significant impacts on having had at least four pre‐natal checkups (0.2 pp; 95% CI: −4.6 to 4.9 pp) or at least three post‐natal checkups for their baby (1.0 pp; 95% CI: −2.8 to 4.7 pp).

**Table 3:** Impacts on MNCH and sanitation‐related outcomes.

	Sample (child age) (months)	*N*	Control mean	Treatment mean	Adjusted impact (95% confidence interval)^a^	*p*‐Value^a^
Pre‐natal and post‐natal care						
Consumed 90 IFA tablets during pregnancy (%)	0–11	3.027	14.2	22.8	8.7 (4.1–13.3)	<0.001***
At least four pre‐natal checkups (%)^b^	0–11	3.042	75.9	76.8	0.2 (−4.6 to 4.9)	0.939
At least three post‐natal checkups for baby within first 4 weeks (%)^b^	1–11	2.616	10.9	11.7	1.0 (−2.8 to 4.7)	0.619
Ever attended nutritional group counselling sessions for pregnant women	0–11	3.040	20.8	29.4	8.4 (2.6–14.1)	0.176
Ever attended nutritional group counselling sessions for caregivers of young children	0–35	9.115	9.7	16.0	6.6 (3.7–9.4)	<0.001***
Child feeding						
EIBF (%)	0‐23	4.596	50.7	54.5	3.6 (−2.0 to 9.2)	0.210
EBF (%)	0–5	1.490	38.0	45.0	8.7 (1.8–15.6)	0.014**
Minimum dietary diversity (%)	6–23	3.115	38.8	40.6	2.3 (−3.0 to 7.7)	0.389
Minimum meal frequency (%)	6–23	3.115	56.1	64.3	8.5 (3.8–13.2)	<0.001***
Minimum acceptable diet (%)	6–23	3.115	23.0	27.8	5.4 (0.9–9.9)	0.019**
Vitamin. A twice yearly (%)	6–35	7.538	30.6	33.5	2.3 (−1.2 to 5.7)	0.190
Growth monitoring						
Weighed monthly (%)	0–5	1.490	75.1	82.0	5.3 (–0.4 to 11.1)	0.069*
Length taken at least once in past year (%)	0–11	3.051	79.3	77.8	–3.2 (–8.9 to 2.5)	0.272
Length taken at least once in past year (%)	12–35	6.039	51.7	65.0	10.8 (5.5–16.1)	<0.001***
Sanitation						
Household has access to an improved latrine (owned or shared) (%)	0–35	9.108	76.6	76.8	0.1 (−4.1 to 4.2)	0.978
Household practiced open defecation in past week (%)	0–35	9.120	34.6	35.0	0.3 (−4.1 to 4.6)	0.909
Child had diarrhoea in past 2 weeks (%)	0–5	1.490	10.1	7.1	–3.5 (–7.9 to 0.8)	0.105
Child had diarrhoea in past 2 weeks (%)	0–35	9.120	13.2	13.8	0.4 (−1.8 to 2.5)	0.751
Child had worms in past 12 months (%)	6–35	7.606	5.7	7.2	1.1 (−0.6 to 3.0)	0.208

*Note*: The village‐level intra‐cluster correlation coefficients ranged from 0.055 to 0.134 for pre‐natal and post‐natal care outcomes, 0.017 to 0.092 for child feeding outcomes, 0.085 to 0.334 for growth monitoring outcomes and 0.022 to 0.157 for sanitation outcomes.

Abbreviations: EBF, exclusive breastfeeding; EIBF, early initiation of breastfeeding; IFA, iron and folic acid.

^a^ Adjusted for district, whether the respondent completed junior secondary school, wealth quintile, mother's age when the sampled child was born, birth order of the sampled child, the sampled child's gender and the baseline outcome at the district level. The design effect of cluster random assignment was accounted for using Stata's cluster option.

^b^ Measured using maternal and child health books when available or through self‐reports otherwise.

There was no significant impact on the percentage of mothers of 0–23‐month‐olds who initiated breastfeeding within 1 hour of birth (3.6 pp; 95% CI: −2.0 to 9.2 pp). However, the project had a positive impact on exclusive breastfeeding; about 45% of 0–5‐month‐olds in the treatment group were exclusively breastfed at endline using the WHO and UNICEF definition (based on feeding in the previous day, World Health Organization WHO, 2008), a statistically significant 8.7pp higher than in the control group (95% CI: 1.8−15.6 pp).

The UNICEF and WHO minimum acceptable diet indicator for children 6−23 months combines standards of dietary diversity and feeding frequency. They recommend a diet that includes at least four of the seven food groups a day to ensure adequate growth (Arimond & Ruel, 2004; World Health Organization [WHO], 2008). The recommended meal frequency depends on the age of the child and whether they are also being breastfed. The project had no impact on minimum dietary diversity for 6–23‐month‐old children (2.3 pp; 95% CI: −3.0 to 7.7 pp) but a significant positive impact on minimum meal frequency (8.5 pp; 95% CI: 3.8−13.2 pp). The criterion for minimum acceptable diet, which combines the dietary diversity and meal frequency indicators, was 5.4 pp in the treatment group relative to the control group (95% CI: 0.9−9.9 pp), driven by the impact on meal frequency. There was no significant impact on the percentage of 6−35‐month‐olds receiving vitamin A twice yearly (2.3 pp; 95% CI: −1.2 to 5.7), the minimum recommended frequency in vitamin A‐deficient settings (WHO, 2011).

In terms of growth monitoring, the impact on the percentage of 0–5‐month‐olds who were weighed monthly was positive but only marginally significant (5.3 pp; 95% CI: −0.4 to 11.1 pp). There was no significant impact on the percentage of 0–11‐month‐olds who had their length measured at least once in the previous year (−3.2 pp; 95% CI: −8.9 to 2.5), but a large positive and significant impact on this outcome for 12–35‐month‐olds (10.8 pp; 95% CI: 5.5−16.1).

### 3.4 Sanitation‐related outcomes

In terms of secondary sanitation‐related outcomes, the project had no impact on whether households had access to an improved latrine (0.1 pp; 95% CI: −4.1 to 4.2 pp) nor on self‐reported open defecation by household members in the previous week (0.3 pp; 95% CI: −4.1 to 4.6 pp) (Table 3). This is consistent with findings from supplementary provider surveys we conducted with sanitarians, which showed little treatment‐control difference in the percentage of villages overseen by the average sanitarian that had achieved ODF status (12.0% vs. 6.8%), despite a large difference in the average percent of villages that were triggered (74.4% vs. 59.1%).

At endline, about 7% of 0–5‐month‐olds in the treatment group had experienced diarrhoea in the previous 2 weeks; this was lower than in the control group but not a statistically significant difference (−3.5 pp; 95% CI: −7.9 to 0.8 pp). The overall incidence of diarrhoea was higher for the full sample of 0–35‐month‐olds, but the impact was also not statistically significant (0.4 pp; 95% CI: −1.8 to 2.5 pp). Nor was there evidence of a significant impact on the incidence of worm infections in the previous 12 months, as reported by caregivers of 6–35‐month‐olds (1.1 pp; 95% CI: −0.6 to 3.0 pp).

## 4 DISCUSSION

Ultimately, the comprehensive package of community‐level interventions in this study failed to reduce stunting in project areas in rural Indonesia, even for the cohort with most project exposure, although the literature suggests that such a package has strong potential to do so. The comprehensive nature of the package of interventions included in the project is an important strength of this study. Among the 10 evidence‐based nutrition interventions that Bhutta et al. (2013) identified as potentially effective in reducing stunting were breastfeeding and complementary feeding promotion in community and facility settings, conditional cash transfers to improve access to care, and the provision of micronutrients, and community‐based delivery platforms. These components were all included in the project, with cash transfers at the community rather than the individual level.

In a subsequent synthesis about the experience of countries that have successfully reduced child stunting at scale, Bhutta et al. (2020) identified several strategies that can support direct nutrition‐related strategies such as maternal nutrition and newborn care, early and exclusive breastfeeding, and complementary feeding practices. A supportive enabling environment, including high‐level political and donor support, is especially critical. Consistent with this recommendation, MCC and the Indonesian government collaborated closely on the design and implementation of this project, which was conceived of and implemented in the context of a national policy focus on reducing stunting. The Ministry of Villages, Disadvantaged Regions, and Transmigration implemented *Generasi* and the Ministry of Health implemented the other project components. The project also included a WASH component to reduce open defecation, which Bhutta et al. (2020) identified as an indirect strategy that has been important in reducing stunting in some but not all countries.

The study had additional strengths. At $120 million, the project was expansive and represented a concerted and well‐resourced effort to reduce stunting. It reached more than 5,000 villages, trained more than 17,000 providers in IYCF and trained almost 7,000 providers in CLTS across 11 of Indonesia's 34 provinces (at the time). We were able to conduct a rigorous randomized controlled trial, albeit not with multiple intervention arms that would have allowed us to parse out the effects of different interventions. The study was also large—it included more than 780 villages and 9,000 households at endline—and spanned a range of geographies across three provinces. The study team was able to devote resources to careful anthropometric measurement, including ensuring inter‐rater reliability through extensive training and high‐quality measurement tools.

The lack of impacts on stunting demonstrates that implementing a multifaceted stunting project at scale in a synchronized manner over a relatively short period can be challenging in practice. For example, the expectation was that *Generasi* would enable communities to use grants to improve the frequency of one‐on‐one services and the frequency of nutritional counselling sessions, which were to be led by providers trained in IYCF through the project. However, providers in some areas only received the IYCF training towards the end of the project. Further, although it was envisioned that trained providers would distribute improved IFA to pregnant women attending the nutritional counselling sessions and in their one‐on‐one counselling sessions with providers, improved IFA tablets were distributed only towards the end of the project due to regulatory approval and procurement delays. Thus far fewer cohorts of women than expected received IFA while benefiting from other project components. Other improved brands also became widely available by the end of the project, so that about 90% of mothers in both treatment and control areas at endline reported consuming an improved brand, although the project‐promoted brand was more common in treatment areas.

For sanitation, the implementer experienced delays and did not meet triggering or ODF targets. Further, there may have been weaknesses in CLTS training content and/or provider capacity to implement it, given the small share of villages that became ODF compared to those that were triggered. It was unlikely that child diarrhoea would decline when open defecation rates remained so high; substantially more intensive interventions might have been required to achieve that (Null et al., 2018; Pickering et al., 2019).

The study identified positive impacts on some secondary, more proximal outcomes related to maternal and child nutrition, including the percentage of mothers consuming the recommended number of IFA pills during pregnancy, exclusive breastfeeding and meal frequency. While the study design did not enable us to disentangle the impact of specific interventions, it is plausible that they were the result of a combination of improved training for community health workers and *Generasi* spending on improving their engagement with community members, such as transportation funds for health workers and clients. However, these impacts were not substantial enough to affect stunting. This might be because the project did not induce large improvements in service access, such as post‐natal checkups or participation in nutritional group counselling sessions. It is also possible that the association between some of these practices and stunting is weak (Prendergast & Humphrey, 2014).

Our findings stress that successfully implementing an integrated package of interventions to reduce child stunting—the approach emphasized by the literature—may be challenging in practice, especially in a typical programme timeframe. Project design needs to consider implementation reality along with best practice—for example, by potentially piloting implementation of multifaceted interventions to be confident that such synchronous implementation is feasible. Alternatively, project components could be phased in more gradually over a longer timeframe, which would require funders to support longer implementation timelines. Overall, even if best practice advocates for interdependent component implementation to reduce child stunting, such advocacy is unlikely to lead to the desired outcomes unless the practicalities of implementation are fully considered.

## AUTHOR CONTRIBUTIONS

Amanda Beatty, Evan Borkum and Clair Null designed the research study. Amanda Beatty, Evan Borkum, Clair Null and Wayan Suriastini developed survey instruments. Wayan Suriastini led fieldwork. Evan Borkum and Amanda Beatty led the analysis. William Leith analysed data. Amanda Beatty and Evan Borkum wrote the paper.

## CONFLICT OF INTEREST STATEMENT

The authors declare no conflicts of interest.

## Data Availability

The data that support the findings of this study are openly available in MCC evidence platform at https://mcc.icpsr.umich.edu/evaluations/index.php/catalog/109.

## 1

**Table A1:** *Generasi* indicators.

	Indicator
1.	Four pre‐natal checkups
2.	Received 90 iron pills during pregnancy
3.	Delivery by trained professional
4a.	Mother received three post‐natal checkups
4b.	Baby received three post‐natal checkups
5.	Complete childhood immunizations
6a.	Vitamin A twice/year, among 12–35‐month‐olds
6b.	Vitamin A twice/year, among 6–35‐month‐olds
7.	0–5‐month‐olds weighed monthly
8.	0–23‐month‐olds weighed monthly
9a.	Pregnant woman ever attended class at village health post
9b.	Expectant father ever attended class with pregnant spouse at village health post
10a.	Mother ever attended class for caregivers of children under 5 at village health post
10b.	Father ever attended class for caregivers of children under 5 at village health post

*Note*: There were 12 *Generasi* indicators in total, which included 10 health‐related indicators and two education‐related indicators. Because this study focused on health‐related indicators, we only show those here.

## References

[mcn13593-bib-0001] Angrist, J. D. , & Pischke, J. S. (2008). Mostly harmless econometrics: An empiricist's companion. Princeton University Press.

[mcn13593-bib-0002] Arimond, M. , & Ruel, M. T. (2004). Dietary diversity is associated with child nutritional status: Evidence from 11 demographic and health surveys. The Journal of Nutrition, 134(10), 2579–2585. 15465751 10.1093/jn/134.10.2579

[mcn13593-bib-0003] Badan Pusat Statistik . (2013). *Population size by province in Indonesia*. https://jatim.bps.go.id/indicator/12/114/2/jumlah-penduduk-menurut-provinsi-di-indonesia.html

[mcn13593-bib-0004] Beatty, A. , Borkum, E. , Leith, W. , Henry, M. , Berends, M. , Null, C. , & Ingwersen, N. (2020). MCC Indonesia nutrition project impact evaluation final report. Final report to the Millennium Challenge Corporation. Mathematica.

[mcn13593-bib-0005] Bhutta, Z. A. , Akseer, N. , Keats, E. C. , Vaivada, T. , Baker, S. , Horton, S. E. , Katz, J. , Menon, P. , Piwoz, E. , Shekar, M. , Victora, C. , & Black, R. (2020). How countries can reduce child stunting at scale: Lessons from exemplar countries. The American Journal of Clinical Nutrition, 112(112), 894S–904S. 32692800 10.1093/ajcn/nqaa153 PMC7487427

[mcn13593-bib-0006] Bhutta, Z. A. , Das, J. K. , Rizvi, A. , Gaffey, M. F. , Walker, N. , Horton, S. , Webb, P. , Lartey, A. , & Black, R. E. (2013). Evidence‐based interventions for improvement of maternal and child nutrition: what can be done and at what cost? The Lancet, 382(9890), 452–477. 10.1016/S0140-6736(13)60996-4 23746776

[mcn13593-bib-0007] Dewey, K. G. (2016). Reducing stunting by improving maternal, infant and young child nutrition in regions such as south Asia: Evidence, challenges and opportunities. Maternal & Child Nutrition, 12(Suppl. 1), 27–38. 27187908 10.1111/mcn.12282 PMC5084734

[mcn13593-bib-0008] De Onis, M. , Onyango, A. W. , Van den Broeck, J. , Chumlea, W. C. , & Martorell, R. (2004). Measurement and standardization protocols for anthropometry used in the construction of a new international growth reference. Food and Nutrition Bulletin, 25(25), S27–S36. 15069917 10.1177/15648265040251S104

[mcn13593-bib-0009] Fink, G. , Günther, I. , & Hill, K. (2011). The effect of water and sanitation on child health: Evidence from the demographic and health surveys 1986–2007. International Journal of Epidemiology, 40(5), 1196–1204. 21724576 10.1093/ije/dyr102

[mcn13593-bib-0010] Headey, D. , & Palloni, G. (2019). Water, sanitation, and child health: Evidence from subnational panel data in 59 countries. Demography, 56(2), 729–752. 30820757 10.1007/s13524-019-00760-y PMC6449314

[mcn13593-bib-0011] Hossain, M. , Choudhury, N. , Adib Binte Abdullah, K. , Mondal, P. , Jackson, A. A. , Walson, J. , & Ahmed, T. (2017). Evidence‐based approaches to childhood stunting in low‐ and middle‐income countries: A systematic review. Archives of Disease in Childhood, 102(10), 903–909. 28468870 10.1136/archdischild-2016-311050 PMC5739821

[mcn13593-bib-0012] Karakochuk, C. , Whitfield, K. , Green, J. , & Kraemer, K. (2017). The biology of the first 1,000 days. CRC Press.

[mcn13593-bib-0013] Kementerian Kesehatan . (2018). *Laporan Nasional Riskesdas*. https://dinkes.babelprov.go.id/sites/default/files/dokumen/bank_data/20181228%20-%20Laporan%20Riskesdas%202018%20Nasional-1.pdf

[mcn13593-bib-0014] Mangasaryan, N. , Arabi, M. , & Schultink, W. (2011). Revisiting the concept of growth monitoring and its possible role in community‐based nutrition programs. Food and Nutrition Bulletin, 32(1), 42–53. 21560463 10.1177/156482651103200105

[mcn13593-bib-0015] Null, C. , Stewart, C. P. , Pickering, A. J. , Dentz, H. N. , Arnold, B. F. , Arnold, C. D. , Benjamin‐Chung, J. , Clasen, T. , Dewey, K. G. , Fernald, L. , Hubbard, A. E. , Kariger, P. , Lin, A. , Luby, S. P. , Mertens, A. , Njenga, S. M. , Nyambane, G. , Ram, P. K. , & Colford, J. M. (2018). Effects of water quality, sanitation, handwashing, and nutritional interventions on diarrhoea and child growth in rural Kenya: A cluster randomised controlled trial. The Lancet Global Health, 6(3), e316–e329. 29396219 10.1016/S2214-109X(18)30005-6 PMC5809717

[mcn13593-bib-0016] Olken, B. A. , Onishi, J. , & Wong, S. (2014). Should aid reward performance? Evidence from a field experiment on health and education in Indonesia. American Economic Journal: Applied Economics, 6(4), 1–34. 25485039 10.1257/app.6.4.1 PMC4254820

[mcn13593-bib-0017] Pickering, A. J. , Null, C. , Winch, P. J. , Mangwadu, G. , Arnold, B. F. , Prendergast, A. J. , Njenga, S. M. , Njenga, S. M. , Rahman, M. , Ntozini, R. , Benjamin‐Chung, J. , Stewart, C. P. , Huda, T. , Moulton, L. H. , Colford, J. M. , Luby, S. P. , Humphrey, J. H. , & (2019). The WASH benefits and SHINE trials: interpretation of WASH intervention effects on linear growth and diarrhoea. The Lancet Global Health, 7(8), e1139–e1146. 31303300 10.1016/S2214-109X(19)30268-2

[mcn13593-bib-0027] Prendergast, A. J. , & Humphrey, J. H. (2014). The stunting syndrome in developing countries. Paediatrics and international child health, 34(4), 250–265. 25310000 10.1179/2046905514Y.0000000158 PMC4232245

[mcn13593-bib-0018] Republic of Indonesia . (2018). National strategy to accelerate stunting reduction 2018‐2024. Republic of Indonesia.

[mcn13593-bib-0019] Spears, D. , Ghosh, A. , & Cumming, O. (2013). Open defecation and childhood stunting in India: An ecological analysis of new data from 112 districts. PLoS ONE, 8(9), e73784. 24066070 10.1371/journal.pone.0073784 PMC3774764

[mcn13593-bib-0020] United Nations Children's Fund (UNICEF) . (2013). Improving child nutrition: The achievable imperative for global progress.

[mcn13593-bib-0021] United Nations Children's Fund (UNICEF) , World Health Organization (WHO) , the World Bank . (2021). Levels and trends in child malnutrition: Key findings of the 2021 edition of the joint child malnutrition estimates. World Health Organization.

[mcn13593-bib-0022] Victora, C. G. , de Onis, M. , Hallal, P. C. , Blössner, M. , & Shrimpton, R. (2010). Worldwide timing of growth faltering: Revisiting implications for interventions. Pediatrics, 125(3), e473–e480. 20156903 10.1542/peds.2009-1519

[mcn13593-bib-0023] Wooldridge, J. M. (2010). Econometric analysis of cross section and panel data. MIT Press.

[mcn13593-bib-0024] World Health Organization (WHO) . (2008). Indicators for assessing infant and young child feeding practices part 1 definitions.

[mcn13593-bib-0025] World Health Organization (WHO) . (2011). Guideline: Vitamin A supplementation in infants and children 6‐59 months of age. 24575452

